# Yolkin, a Polypeptide Complex from Egg Yolk, Affects Cytokine Levels and Leukocyte Populations in Broiler Chicken Blood and Lymphoid Organs after In Ovo Administration

**DOI:** 10.3390/ijms242417494

**Published:** 2023-12-14

**Authors:** Marianna Szczypka, Magdalena Lis, Maciej Kuczkowski, Kamila Bobrek, Aleksandra Pawlak, Aleksandra Zambrowicz, Andrzej Gaweł, Bożena Obmińska-Mrukowicz

**Affiliations:** 1 Department of Pharmacology and Toxicology, Faculty of Veterinary Medicine, Wrocław University of Environmental and Life Sciences, Norwida 31, 50-375 Wrocław, Poland; magdalena.lis@upwr.edu.pl (M.L.); aleksandra.pawlak@upwr.edu.pl (A.P.); bozena.obminska-mrukowicz@upwr.edu.pl (B.O.-M.); 2 Department of Epizootiology and Clinic of Birds and Exotic Animals, Faculty of Veterinary Medicine, Wrocław University of Environmental and Life Sciences, Pl. Grunwaldzki 45, 50-366 Wrocław, Poland; maciej.kuczkowski@upwr.edu.pl (M.K.); kamila.bobrek@upwr.edu.pl (K.B.); andrzej.gawel@upwr.edu.pl (A.G.); 3 Department of Functional Food Products Development, Faculty of Biotechnology and Food Science, Wrocław University of Environmental and Life Sciences, Chełmońskiego 37, 51-640 Wrocław, Poland; aleksandra.zambrowicz@upwr.edu.pl

**Keywords:** yolkin, chicken immunity, cytokines, leukocyte subsets

## Abstract

Yolkin is a polypeptide complex isolated from hen egg yolk that exhibits immunomodulating properties. The aim of the present study was to determine whether in-ovo-delivered yolkin affects leukocyte populations and cytokine levels in broiler chickens. The experiment was carried out on eggs from Ross 308 broiler breeder birds. Yolkin was administered in ovo on the 18th day of incubation, once, at the following three doses: 1, 10, or 100 µg/egg. The immunological parameters were assessed in 1-, 7-, 14-, 21-, 28-, 35-, and 42-day-old birds kept under farming conditions and routinely vaccinated. The leukocyte populations were determined in the thymus, spleen, and blood. The cytokine (IL-1β, IL-2, IL-6, and IL-10) levels were determined in the plasma of the broiler chickens. Each experimental group included eight birds. The most pronounced effect of yolkin was an increase in the population of T cells, both CD4^+^ and CD8^+^, mainly in the blood. This effect on the lymphocyte subsets may be valuable regarding chicken immune responses, mainly against T-dependent antigens, during infection or after vaccination.

## 1. Introduction

According to the “One health” concept, to minimize the risk to animal health or public safety caused by the development of antimicrobial resistance, the use of antimicrobial drugs should be reduced both in humans and animals [1,2]. Therefore, antibiotics should be administered according to the concept of rational and prudent use. Nowadays, in the countries of the European Union, the legislation concerning the use of antibiotics in animals is much more stringent, for example, antimicrobial drugs generally should not be used in animals for prophylaxis [3]. The reduced use of antibiotics not only decreases the emergence of resistant bacteria, but also lowers the risk of drug side effects in animals and drug residues in food-producing animals.

Due to this, there is an urgent need to search for new antimicrobial approaches to minimize the high risk of infection in broiler chickens under farming conditions [4,5]. One of the solutions is the use of bacteriophages, which are capable of specifically reducing, or even eliminating, pathogenic bacteria [6,7,8,9]. The other goal is to limit the susceptibility of the broilers to infections. This can be achieved by applying immunomodulating substances that improve the birds’ immune status. For this purpose, bioactive substances, such as probiotics, prebiotics, and synbiotics [4,10,11,12], or plant compounds [5,13,14,15,16], are used in broilers as dietary supplementation. These substances of natural origin can not only effectively promote and maintain gut health, but also positively affect the immune system. 

The beneficial effects of bioactive substances on the immune system and growth performance of the broilers were observed not only after their use as feed additives, but also after in ovo administration [17,18,19,20]. This route of administration facilitates the supply of the immunomodulating substances as early as possible and eliminates the impact of different feed intake by individual birds. A very important aspect is also the possibility of automatic in ovo injection, which facilitates the use of this method in field conditions [18]. It has been shown that in-ovo-delivered substances may affect the immune system, including the morphology of the central and peripheral lymphoid organs, as well as lymphocyte populations [17,20,21,22,23].

One of the immunomodulating substances is yolkin, a vitellogenin-derived polypeptide complex contained in egg yolk plasma accompanying the main avian immunoglobulin IgY [24,25,26]. The protein profile of yolkin depends on different factors, such as bird species, nutrition, habitat, exposure to pathogens, and others [26]. However, it is well worth noting that, regardless of the protein pattern, yolkin exhibits similar biological properties [26]. It has been shown that yolkin, when used as an exogenous substance, presents immunoregulatory and neuroprotective activity [24,25,27,28,29,30,31]. As an immunomodulator, yolkin is capable of inducing the secretion of several cytokines (IL-1β, IL-6, IL-10, and TNF-α) and has various effects on nitric oxide (NO) production [24,25,27,28]. Moreover, our previous study, conducted in vitro and in vivo, showed that yolkin promotes the maturation of immunocompetent cells (T and B lymphocytes) and stimulates the humoral immune response [29]. Still, the mechanism of the immunomodulatory effect of yolkin is not fully understood. In addition to its immunomodulatory properties, yolkin possesses neuroprotective activity [30,31]. In a study conducted on rats, Lemieszewska et al. [30] demonstrated the pro-cognitive action of yolkin, which mitigated the behavioral symptoms of aging and improved cognitive functions. The authors stated that yolkin may be a promising substance for the prevention and treatment of neurodegenerative disorders, because it may inhibit the progression of dementia [30]. The molecular mechanism of this neuroprotective activity of yolkin was examined by Kazana et al. [31] in a study conducted in vitro in a rat pheochromocytoma cell line (PC12) and fetal rat hippocampal cell line (H19-7). Yolkin was found to upregulate the phosphorylation of the cAMP response element-binding protein (CREB) [31]. Moreover, yolkin increased the expression/production of important neurotrophic factors, such as brain-derived neurotrophic factor (BDNF) and carboxypeptidase E/neurotrophic factor-α1 (CPE/(NF-α1)) [31].

The aim of this study was to evaluate the immunotropic action of in-ovo-delivered yolkin on the maturation of the immune system with regard to cytokine production and leukocyte population in the lymphoid organs, as well as in the blood, during the whole life of routinely vaccinated broiler chickens kept under farming conditions. 

## 2. Results

All of the data and statistical differences between all of the groups are presented in the tables and figures. The data were analyzed using one-way analysis of variance (ANOVA), followed by Tukey’s post hoc test, to show the differences between all of the experimental groups. However, to simplify the data presentation, the description of the results contains only the changes that are statistically significant compared with the control group. 

### 2.1. Effects of Yolkin on the Thymocyte Populations

Yolkin, at a dose of 100 µg/egg, transiently increased (in 7-day-old birds) both the percentage and total count of CD4^−^CD8^−^ thymocytes. Yolkin, administered in ovo at 1 and 10 µg/egg, did not affect the subpopulation of double-negative (CD4^−^CD8^−^) thymocytes (Table S1).

Yolkin reduced the subset of double-positive CD4^+^CD8^+^ thymocytes. At 100 µg/egg, it decreased the percentage of these cells in the 7- and 21-day-old birds; at 10 µg/egg, it lowered their total count in the 14- and 35-day-old broilers; and, at 1 µg/egg, it reduced both the percentage and the total count in the 35-day-old birds (Table 1).

As compared with the control group, none of the tested doses of yolkin changed the subpopulations of the single-positive (CD4^+^ and CD8^+^) thymocytes or the CD4^+^/CD8^+^ ratio in the thymus (Tables S2–S4).

### 2.2. Effects of Yolkin on the Populations of Splenic Leukocytes

Yolkin, regardless of the dose used, reduced both the percentage and the total count of the Bu-1a^+^ cells in the spleen of the 14-day-old birds. Additionally, a drop in the percentage of Bu-1a^+^ cells was noticed after a dose of 1 µg/egg in the 35-day-old birds (Table 2).

An increase in the percentage of T lymphocytes (CD3^+^ cells) was noticed in the 1-day-old chicks, regardless of the dose used. In the same birds, a dose of 10 µg/egg boosted the total count of T splenocytes. Yolkin, at a dose of 10 µg/egg, decreased the percentage of CD3^+^ splenocytes in the 28-day-old birds. A dose of 1 µg/egg increased the percentage of CD3^+^ cells in the 35-day-old birds. In the 42-day-old birds, a rise in the total count of T splenocytes was noticed after the doses of 10 µg/egg and 1 µg/egg (Table 3).

Yolkin, at 10 µg/egg, enhanced the subset of CD4^+^ splenocytes as follows: the percentage in the 1-day-old chicks and the total count in the 42-day-old birds. Yolkin, at 1 µg/egg, amplified both the percentage and the total count of CD4^+^ splenocytes in the 35- and 42-day-old broilers (Table 4). 

An increase in the percentage of CD8^+^ splenocytes was noticed at 100 and 10 µg/egg in the 1- and 42-day-old broilers. Yolkin, regardless of the dose used, increased the total count of CD8^+^ cells in the 42-day-old birds (Table 5).

Yolkin, at 100 and 10 µg/egg, did not change the CD4^+^/CD8^+^ ratio. A rise in the CD4^+^/CD8^+^ ratio was noticed after a dose of 1 µg/egg in the 35- and 42-day-old birds (Table S5).

At 100 µg/egg, yolkin transiently lowered (in the 14-day-old broilers) and then increased (in the 35-day-old broilers) both the percentage and the total count of splenic macrophages (KUL01^+^ cells). The variable effects on the population of the splenic macrophages were observed after a dose of 10 µg/egg as follows: an enhanced percentage in the 7- and 28-day-old birds, a reduced percentage in the 1-day-old chicks, and a drop in both the percentage and the total count in the 14-day-old birds. Yolkin, at 1 µg/egg, did not affect the population of macrophages in the spleen (Table 6).

### 2.3. Effects of Yolkin on the Populations of Blood Leukocytes 

Yolkin, administered in ovo at a dose of 10 µg/egg, transiently decreased the percentage of Bu-1a^+^ cells in the blood of the 1-day-old chicks. An increase in the Bu-1a^+^ population was observed after the administration of yolkin at a dose of 100 µg/egg in the 7-day-old birds (percentage and total count) and at 1 µg/egg in the 35-day-old birds (total count) (Table 7).

An increase both in the percentage and the total count of T lymphocytes (CD3^+^ cells) was noticed in the blood of the 1-day-old chicks after in ovo yolkin administration at 10 µg/egg. The same dose augmented the total count of T cells in the 14-day-old birds. In the 42-day-old chickens, an increase in the total count of T cells was observed at the doses of 100 and 1 µg/egg (Table 8).

A rise in the total count of CD4^+^ T lymphocytes was noticed in the 1-day-old and 14-day-old birds after the dose of 10 µg/egg; in the 28-day-old birds after a dose of 100 µg/egg; and in the 42-day-old birds after a dose of 1 µg/egg. However, yolkin did not affect the percentage of CD4^+^ cells, as compared with the control group (Table 9).

Yolkin, administered in ovo, augmented the subsets of the CD8^+^ T cells. An increase in the percentage of the CD8^+^ cells was noticed after a dose of 100 µg/egg in the 42-day-old birds; after a dose of 10 µg/egg in the 1-day-old chicks; and after a dose of 1 µg/egg in the 14- and 28-day-old birds. A rise in the total count of CD8^+^ T lymphocytes was observed in the 1-day-old chicks after all of the tested doses of yolkin; in the 14-day-old birds after a dose of 10 µg/egg; and in the 42-day-old birds after a dose of 100 µg/egg (Table 10).

There was no change in the CD4^+^/CD8^+^ ratio at a yolkin dose of 100 µg/egg and 10 µg/egg. A dose of 1 µg/egg reduced this ratio in the 28-day-old birds (Table S6).

Yolkin, at a dose of 1 µg/egg, increased (in the 1-day-old chicks) and then decreased (in the 7-day-old birds) both the percentage and the total count of monocytes (KUL01^+^ cells). The decrease in the percentage of monocytes was noticed in the 7-day-old birds after a dose of 10 µg/egg. In-ovo-delivered yolkin, at a dose of 100 µg/egg, did not affect either the percentage or the total count of monocytes (Table 11).

### 2.4. Effects of Yolkin on the Cytokine Levels

**Figure 1:**
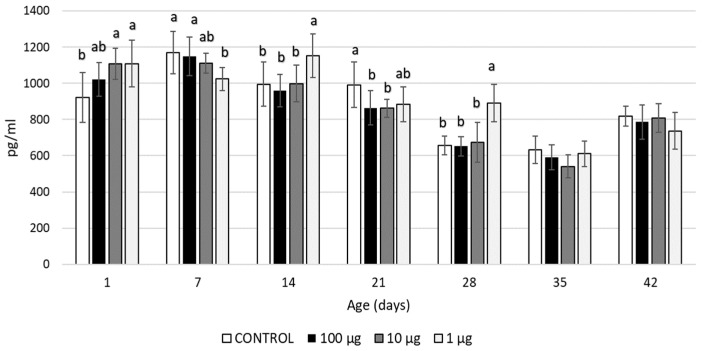
IL-1β levels (pg/mL) in the blood of broiler chickens after in ovo injection of yolkin. The values are presented as mean ± SD; n = 8. The values within the same age group with no common superscript letter differ significantly (*p* < 0.05).

Yolkin, administered in ovo at a dose of 1 µg/egg, increased the production of IL-1β in the 1-, 14-, and 28-day-old birds, with a transient decrease observed in the 7-day-old broilers. Yolkin, at a dose of 10 µg/egg, boosted the IL-1β production in the 1-day-old chicks and reduced it in the 21-day-old birds. After a dose of 100 µg/egg, a decrease in the IL-1β level was noticed in the 21-day-old birds (Figure 1).

**Figure 2:**
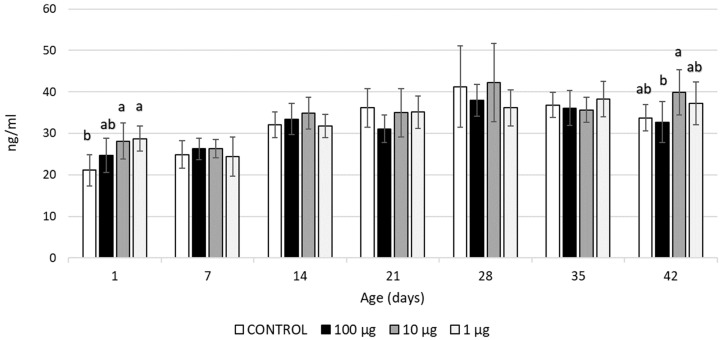
IL-2 levels (ng/mL) in the blood of broiler chickens after in ovo injection of yolkin. The values are presented as mean ± SD; n = 8. The values within the same age group with no common superscript letter differ significantly (*p* < 0.05).

An increase in the production of IL-2 was noticed in the 1-day-old chicks after administering yolkin at 1 µg/egg and 10 µg/egg. Yolkin, at a dose of 100 µg/egg, did not change the IL-2 levels (Figure 2).

A drop in IL-6 production in the 35-day-old birds was observed after a yolkin dose of 100 µg/egg (Figure S1).

Finally, yolkin at 1 µg/egg increased the IL-10 level in the 35-day-old boilers (Figure S2).

## 3. Discussion

As far as we know, our study is the first to demonstrate that in-ovo-delivered yolkin, a polypeptide complex from hen egg yolk accompanying immunoglobulin Y (IgY), may affect the cytokine production and immune cell phenotype in the lymphoid organs and blood of broiler chickens.

The available literature contains limited information on the immunotropic effects of yolkin, especially from in vivo studies. The possible mechanism of the immunotropic action of yolkin was examined by Obmińska-Mrukowicz et al. [29]. Regarding B cells, the same authors showed that yolkin knocked out the expression of the gamma IL-2 receptor (IL-2R) subunit in WEHI 231, a murine immature B cell line, which may indicate the promotion of B cell maturation [29]. In the T cells, yolkin stimulated the expression of all of the families of the mitogen-activated protein kinases (MAPKs) ERK, JNK, and p38 in Jurkat, a human immature T cell line [29]. Similarly, in the macrophages, yolkin upregulated the expression of cytokine mRNA through the activation of MAPKs (ERK and JNK), as well as the phosphoinositide 3-kinase/protein kinase B (PI3K/Akt) signaling pathway [27,32]. 

In the present study, yolkin evoked more pronounced changes in the content of T cells. In the thymus, the primary lymphatic organ responsible for the maturation of T lymphocytes, we determined the subsets of T cells (double-negative, double-positive, and single-positive cells) and found a decrease in the subset of double-positive (CD4^+^CD8^+^) thymocytes after yolkin administration. This effect is in accordance with the study of Obmińska-Mrukowicz et al. [29] conducted on mice, who reported that yolkin, administered intraperitoneally (i.p.) five times at a dose of 0.1 or 1 mg/kg b.w., lowered the percentage of double-positive cells in the thymus, with an accompanying increase in the subsets of single-positive thymocytes, both CD4^+^ and CD8^+^ cells. The authors concluded that these results suggested an induction of T cell maturation in the thymus. In our study, the subsets of single-positive thymocytes were unchanged. However, we observed a rise in the population of cells bearing the pan T cell marker (CD3), both in the blood and in the spleen, wherein an increase occurred in both subpopulations of the T cells, helper (CD4^+^), and suppressor/cytotoxic (CD8^+^) cells. In summary, the above-mentioned observations regarding the T cells in the thymus and peripheral content in the blood and spleen may suggest accelerated T cell maturation and an increased output of T cells into the periphery by in-ovo-delivered yolkin.

In our research, these effects of yolkin on T cell populations, especially in the spleen, showed a similar pattern with the effects on IL-2 production, as an increase in the IL-2 level and T cell populations was observed in the 1-day-old birds and at the end of fattening, i.e., in the 35/42-day-old broilers. IL-2, as a growth factor for T cells, plays an essential role in the immune system, primarily via direct effects on the T cells during their maturation in the thymus, as well as on the functioning of mature CD4^+^ and CD8^+^ T cells [33]. Thus, our findings have suggested that the impact of yolkin on the T cell populations may have partly resulted from its stimulating effect on IL-2 production. 

In the previous study conducted in vivo on mice, Obmińska-Mrukowicz et al. [29] found that yolkin boosted the content of B cells in the spleen and mesenteric lymph nodes, with an accompanying decrease in the T cell population. These effects were dose-dependent, i.e., the lower the yolkin dose, the stronger the effect. In our study, we witnessed a different situation, that is, a rise in T cell content, especially in the blood, and only a slight impact of yolkin on the content of Bu-1a^+^ cells. It is difficult to explain the reason for these differences, as there are many variables that differentiate these experiments, such as the animal species, the route of administration, the tested doses, etc. The cell surface marker Bu-1 is expressed mainly in early and mature B cells [34]. This marker is also found in monocyte/macrophage cell lines. However, they are the minority within the Bu-1+ cell population [34].

Regarding cytokines, there are literature reports on the stimulatory effects of yolkin on the secretory activity of cells from monocyte–macrophage cell lineage in vitro [24,25,27,28,29,35]. In such conditions, yolkin enhanced the production of TNF-α, IL-1β, IL-6, and IL-10 in human whole blood cell cultures [24,25], as well as nitric oxide (NO) release by the murine macrophage cell line J774.2 [25] and murine-bone-marrow-derived macrophages (BMDM) [27]. Moreover, similar effects were provided by a potential precursor of yolkin, recombinant YGP40, which increased TNF-α, IL-10, and NO synthesis in human whole blood cells and murine-bone-marrow-derived macrophages [35]. On the other hand, Obmińska-Mrukowicz et al. [29] reported that yolkin administered to mice diminished ex vivo NO synthesis by peritoneal macrophages stimulated with lipopolysaccharide (LPS). 

The current results differed from the stimulatory effects mentioned above, because, in this study, the impact of yolkin on the content and secretory activity of monocytes/macrophages was ambiguous. However, our findings partially confirm those of the in vitro study conducted by Kazana et al. [32], who reported that yolkin may be a regulator of macrophage function, mainly of the M1 phenotype. There are two main types of macrophages, the classically activated (M1) and the alternatively activated (M2) phenotypes, and they differ, among other things, in the type of cytokines released. M1 macrophages produce high amounts of pro-inflammatory cytokines, such as IL-1β, IL-6, and TNF-α, while M2 macrophages are capable of releasing anti-inflammatory cytokines, such as IL-10 and TGF-β [36,37]. In the present study, the in-ovo-delivered yolkin did not actually affect the production of IL-10 and IL-6, as in the case of both cytokines, where only a single significant change in the 35-day-old birds was noticed. However, yolkin changed the IL-1β level in variable ways depending on the dose used and the age of the birds. Some of these changes correlated with the impact of yolkin on the population of monocytes in the blood, for example, the dose of 1 µg/egg increased the IL-1β production in the 1-day-old birds and decreased it in the 7-day-old broilers. In the same bird groups, the content (both the percentage and the total count) of monocytes was higher in the 1-day-old birds and lower in the 7-day-old broilers. These observations indicated that the impact of yolkin on the IL-1β level may be partially due to its effect on the number of cells releasing this cytokine. Kazana et al. [32] showed that yolkin is able to activate the PI3K/Akt pathway. This signaling pathway plays a key role in the regulation of the activation and functions of the monocytes/macrophages, including the migration of these cells [38,39]. The yolkin effect on the KUL01^+^ cell populations in the 1-day-old chicks (an increase in the blood and a decrease in the spleen) and in the 7-day-old birds (the opposite effect) may result from this stimulating impact of yolkin on the PI3K/Akt pathway.

In summary, in-ovo-delivered yolkin may be a promising approach for enhancing the immune system functioning of broiler chickens. The present research has shown that the most pronounced effect of yolkin was an increase in the content of T cells, both CD4^+^ and CD8^+^. This influence was observed both in the blood and the spleen, especially after the doses of 1 and 10 µg/egg. The described impact may be valuable during infection or after vaccination regarding chicken immune responses, mainly against T-dependent antigens. However, the evaluation of yolkin activity in these aspects requires further detailed research in order to clarify its immunotropic effect.

## 4. Materials and Methods

The experiment was carried out in accordance with the requirements of the European Union, with the consent of the Local Ethics Committee for Animal Experiments, Wrocław, Poland (the opinion number 062/2019 and permission number 082/2019).

### 4.1. Yolkin Preparation

The preparation of the yolkin was performed according to Polanowski et al. [24], with minor modifications. The hen eggs were individually broken, and the yolks were diluted with 9 volumes of distilled water (4 °C), mixed, and the pH of the suspension was then adjusted to 5.0 with 1 M HCl. The suspension was allowed to stand in a freezer (–20 °C) for 24 h. After slow thawing, the yolk suspension was centrifuged (9000× *g* for 30 min at 4 °C), and the supernatant was supplemented with charcoal to a final concentration of 0.01%. Then, the pH of the mixture was readjusted to 4.0, under slow stirring for 30 min, and centrifuged again as above. After that, the pH of the supernatant was adjusted to 9.0 (1 M NaOH). Protein precipitation was carried out at 4 °C overnight by the addition of solid ammonium sulfate to obtain a final saturation of 40%. The mixture was then stirred gently overnight and centrifuged as above. The resulting pellet was resuspended in a small volume of water, followed by dialysis against water (24 h) to remove salt, and then against two changes of 100 mM potassium phosphate buffer, pH 7.2 (24 h). Then, the protein preparation, clarified by centrifugation, was chromatographed on a Sephacryl S-100 HR column (2.5 cm × 100 cm), equilibrated with the same buffer (v = 1 mL/min). The fractions (4 mL) were collected for 8 h, and their absorbance was measured at 280 nm. The chromatographic profile showed a main peak corresponding to immunoglobulin Y and a small peak with a longer retention time than that for IgY corresponding to low-molecular-weight proteins and polypeptides (yolkin). The yolkin fractions were pooled, dialyzed against water, and lyophilized.

#### SDS-PAGE Analysis

The SDS/polyacrylamide (10%) slab gel was prepared by the use of TXG Fast Cast Acrylamide solutions (Bio-Rad, Hercules, CA, USA). The protein samples (15 μg) were diluted with the buffer containing a reducing reagent, heated at 100 °C for 2 min, and loaded onto the gel slabs. At the end of the analysis, the gel slab was stained with Coomassie G-250. 

**Figure 3:**
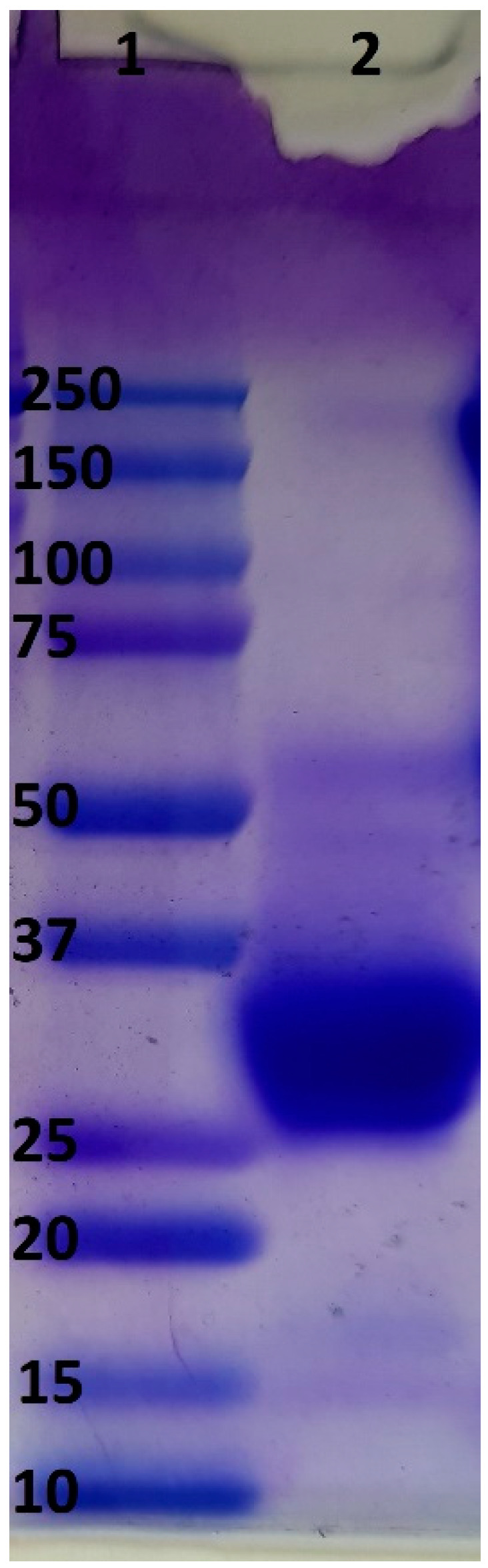
SDS-PAGE of the yolkin preparation (1). MW: molecular weight markers 10-250 kDa (2).

As visible on the electropherogram, the yolkin isolated from hen’s egg yolk was composed of numerous proteins, with a molecular weight (MW) between 25 kDa and 37 kDa (Figure 3). These results are confirmed in the literature. Yolkin isolated from hens’ eggs has been described as a complex composed of the main proteins of 16-35 kDa MW [24,28]. However, the profiles of yolkin obtained by other authors vary, and the presence and proportion of polypeptides greatly depend on the time when the eggs were collected, or even on the pen type, the isolation method, and other factors [24,26,28].

### 4.2. Animals and Treatment

The experiment was carried out on Ross 308 broiler breeder bird (30 weeks of age) eggs (56–59 weight), incubated under large-scale commercial hatchery conditions (Zakład Wylęgu Drobiu S.c. T. Sztuder i A. Sztuder ZWD Wijewo, Wijewo, Poland) in Petersime BioS12SOX incubators (Petersime, Zulte, Belgium). On the 18th day of the incubation period, 600 randomly chosen embryonated eggs were divided into 4 groups (150 eggs/group), i.e., a control group and 3 groups that received yolkin in 1 of the tested doses. The solutions of yolkin were prepared by dissolving yolkin in 0.9% saline solution in that proportion to have 100, 10, or 1 μg of yolkin in the volume 0.05 mL of 0.9% sterile saline solution. The control group was injected with 0.05 mL of 0.9% sterile saline solution/egg, and the next groups were injected as follows: 100 μg of yolkin in 0.05 mL/ egg, 10 μg of yolkin in 0.05 mL/egg, and 1 μg of yolkin in 0.05 mL/egg. The injections were made into the amnion using Innovatec Inovo (Innovatec Hatchery Automation BV, Asperen, The Netherlands). In industrial conditions, the 18th day of the incubation period is the most sensible time point for in ovo administration [18,40]. After the injections, the eggs were marked and moved to separate hatcher baskets in the Petersime BioS4H hatchers (Petersime, Zulte, Belgium). There were no differences in hatchability between the experimental groups, and the hatchability values (87–88%) were typical for breeder flock of this age. 

After hatching, the chickens were transported to poultry houses at a commercial farm. The experimental groups were as follows: the control group, the birds that received yolkin at a dose of 100 µg/egg, the birds that received yolkin at a dose of 10 µg/egg, and the birds that received yolkin at a dose of 1 µg/egg. Each group stayed within boxes separated with wire mesh within an area of 20 m^2^ each, with access to a watering and feeding system. The birds were reared under identical environmental conditions in accordance with the Aviagen Management Book (2018) recommendations [41] and fed commercial diets (starter, grower, and finisher), according to the animal welfare recommendations) [42]. The nutritional composition of the starter, grower, and finisher diets is given in Table S7. The birds had access to feed and water ad libitum and were provided with adequate husbandry conditions, with continuous monitoring of humidity, litter, ventilation, etc., [41]. The birds were vaccinated as follows: on day 1 against Marek disease (Poulvac Marek HVT, Zoetis, Gerona, Spain), infectious bronchitis (Poulvac IB Primer, Zoetis, Gerona, Spain), and Newcastle disease (Poulvac NDW, Zoetis, Gerona, Spain); on day 10 against infectious bronchitis (Nobilis IB 4-91, Intervet International B.V., Boxmeer, The Netherlands); and on day 21 against infectious bursal disease (Avishield IBD INT, Genera Inc., Kalinovica, Croatia).

### 4.3. Measurements

Randomly chosen birds (8 birds from each group) were sacrificed on the 1st, 7th, 14th, 21st, 28th, 35th, and 42nd day of their life. The birds were slaughtered according to Annex IV of Directive 2010/63/EU of the European Parliament and of the Council (by decapitation or cervical dislocation, with/without a percussive blow to the head, according to their age and body weight). Then, their blood was taken from the cervical vein into tubes containing EDTA (Equimed, Kraków, Poland) and their lymphoid organs (thymus and spleen) were removed.

The cytokine (IL-1β, IL-2, IL-6, and IL-10) plasma levels and leukocyte populations in the blood and spleen were determined in the 1-, 7-, 14-, 21-, 28-, 35-, and 42-day-old birds. The lymphocyte subsets in the thymus were determined in the 7-, 14-, 21-, 28-, 35-, and 42-day-old broilers. The plasma samples for cytokine analyses were stored at –80 °C.

#### 4.3.1. Determination of Leukocyte Population in the Blood and Lymphoid Organs

The blood was layered on the isolation mixture Histopaque^®^-1077 (Sigma–Aldrich, St. Louis, MO, USA) at a 1:1 ratio. The isolation of the leukocytes was performed as described previously [19]. The lymphoid organs were placed in Petri dishes in a sterile, ice-cold phosphate-buffered saline solution (PBS) (Institute of Immunology and Experimental Therapy, Wrocław, Poland). Then, the cells were released from the organs by a passage through nylon mesh. The cell suspensions were layered on the isolation mixture Histopaque^®^-1077 at a 1:1 ratio. After centrifugation (3000× *g*, 15 min, 4 °C), the leukocytes from both the blood and the lymphoid organs were collected from the interphase, washed twice with 4 °C PBS with 1% bovine serum albumin (BSA) (Sigma–Aldrich, St. Louis, MO, USA), centrifuged (380× *g*, 7 min, 4 °C), and then suspended in PBS.

The leukocyte suspensions were incubated (30 min, 4 °C) with monoclonal anti-chicken antibodies at concentrations recommended by the manufacturer (SouthernBiotech, Birmingham, AL, USA). The following monoclonal antibodies were used in this experiment: Mouse Anti-Chicken Bu-1a-PE, Mouse Anti-Chicken CD3-FITC, Mouse Anti-Chicken CD4-PE, Mouse Anti-Chicken CD8a-APC, and Mouse Anti-Chicken Monocyte/Macrophage-FITC (KUL01). The experiment also included staining with the following appropriate isotype controls: Mouse IgG1-FITC, Mouse IgG1-PE, and Mouse IgG1-APC. 

After incubation, the cells were washed two times with ice-cold PBS and were centrifuged (380× *g*, 7 min, 4 °C). Then they were fixed in PBS with 2% formaldehyde (Chempur, Piekary Śląskie, Poland). The fluorescence was measured using a flow cytometer (BD FACSCalibur, BD Biosciences, San Jose, CA, USA). The distribution of leukocyte markers was analyzed using CellQuest 3.1f. Pro software (Becton Dickinson, San Jose, CA, USA). The percentage and total count of the leukocytes and the ratio of the CD4^+^/CD8^+^ lymphocytes were determined.

The cells were incubated with the following antibodies: the thymocytes with anti-CD3, anti-CD4, and anti-CD8 antibodies; and the splenocytes and blood cells with anti-Bu-1a, anti-CD3, anti-CD4, anti-CD8, and anti-Monocyte/Macrophage (KUL01) antibodies. 

#### 4.3.2. Determination of Cytokine (IL-1β, IL-2, IL-6, and IL-10) Levels in the Plasma 

The blood samples were taken as described above. First, they were mixed for 20 min and centrifugated (3000× *g*, 30 min, 4 °C). Then, the supernatants were collected and stored at –70 °C. The levels of IL-1β (pg/mL), IL-2 (ng/mL), IL-6 (pg/mL), and IL-10 (pg/mL) were determined using the following commercial ELISA kits: Chicken Interleukin-1β (Qayee-Bio, Shanghai, China), Chicken Interleukin-2 (Qayee-Bio, Shanghai, China), Chicken Interleukin-6 (Qayee-Bio, Shanghai, China), and Chicken Interleukin-10 (Qayee-Bio, Shanghai, China), respectively, according to the manufacturer’s instructions (at the recommended wavelength), using a microplate reader Tecan Spark 10M (Tecan, Switzerland). Each sample was tested in duplicate.

#### 4.3.3. Statistical Analysis 

The data obtained in this experiment were analyzed using one-way analysis of variance (ANOVA), followed by Tukey’s post hoc test for multiple comparisons. The differences were classified as statistically significant at *p* < 0.05. 

## Figures and Tables

**Table 1:** The percentage and total count of CD4^+^CD8^+^ cells in the thymus of broiler chickens after in ovo injection of yolkin. The values are presented as mean ± SD; n = 8. The values in the same line with no common superscript letter differ significantly (*p* < 0.05).

Age (Days)		CONTROL	100 µg/Egg	10 µg/Egg	1 µg/Egg
1		not tested	not tested	not tested	not tested
7	%	87.91 ^a^ ± 1.19	82.00 ^b^ ± 3.37	87.64 ^a^ ± 1.58	86.37 ^a^ ± 2.18
	Total count (×10^6^)	306.29 ± 44.34	287.56 ± 24.74	281.65 ± 27.73	317.54 ± 46.01
14	%	85.97 ± 3.04	81.17 ± 6.13	84.66 ± 2.85	85.60 ± 0.44
	Total count (×10^6^)	607.50 ^ab^ ± 100.96	504.36 ^bc^ ± 31.33	492.51 ^c^ ± 58.09	631.83 ^a^ ± 112.84
21	%	86.85 ^a^ ± 2.04	82.10 ^b^ ± 2.95	84.89 ^ab^ ± 2.05	83.76 ^ab^ ± 3.22
	Total count (×10^6^)	822.35 ± 121.37	761.00 ± 112.42	878.84 ± 158.51	812.86 ± 160.36
28	%	83.95 ± 1.85	83.47 ± 2.07	83.04 ± 3.33	82.36 ± 3.39
	Total count (×10^6^)	1396.30 ± 147.99	1391.85 ± 334.99	1405.29 ± 248.96	1242.74 ± 305.34
35	%	84.50 ^a^ ± 1.75	83.11 ^ab^ ± 2.81	83.14 ^ab^ ± 0.77	78.75 ^b^ ± 5.95
	Total count (×10^6^)	1840.64 ^a^ ± 366.42	1682.16 ^ab^ ± 219.42	1430.21 ^bc^ ± 294.12	1236.59 ^c^ ± 171.59
42	%	84.45 ± 3.61	85.09 ± 2.55	83.28 ± 3.46	84.09 ± 3.42
	Total count (×10^6^)	1979.72 ± 466.87	2023.75 ± 531.18	1818.99 ± 257.32	1645.80 ± 339.46

**Table 2:** The percentage and total count of Bu-1a^+^ cells in the spleen of broiler chickens after in ovo injection of yolkin. The values are presented as mean ± SD; n = 8. The values in the same line with no common superscript letter differ significantly (*p* < 0.05).

Age (Days)		CONTROL	100 µg/Egg	10 µg/Egg	1 µg/Egg
1	%	60.86 ± 5.58	57.63 ± 4.48	55.97 ± 3.10	57.47 ± 2.43
	Total count (×10^6^)	3.29 ± 0.88	3.00 ± 0.55	2.76 ± 0.84	2.70 ± 0.68
7	%	39.64 ± 4.50	37.63 ± 5.00	39.54 ± 3.01	40.08 ± 2.02
	Total count (×10^6^)	32.77 ± 4.17	27.51 ± 5.52	27.91 ± 7.27	28.85 ± 10.43
14	%	41.15 ^a^ ± 4.18	32.08 ^b^ ± 8.68	29.82 ^b^ ± 4.64	33.36 ^b^ ± 3.39
	Total count (×10^6^)	120.27 ^a^ ± 33.50	80.13 ^b^ ± 22.31	63.60 ^b^ ± 14.67	85.91 ^b^ ± 21.93
21	%	47.42 ± 3.29	41.59 ± 5.41	46.14 ± 6.87	44.29 ± 3.52
	Total count (×10^6^)	211.76 ± 43.73	175.10 ± 49.46	189.12 ± 62.89	193.33 ± 36.07
28	%	43.40 ^ab^ ± 2.54	40.89 ^b^ ± 5.70	45.95 ^a^ ± 2.75	43.30 ^ab^ ± 2.69
	Total count (×10^6^)	223.75 ± 26.14	216.78 ± 41.15	224.86 ± 31.50	203.84 ± 44.94
35	%	51.33 ^ab^ ± 3.04	53.56 ^a^ ± 4.81	46.73 ^bc^ ± 3.92	41.89 ^c^ ± 3.56
	Total count (×10^6^)	712.16 ^ab^ ± 58.25	800.43 ^a^ ± 184.77	664.15 ^ab^ ± 151.98	519.32 ^b^ ± 138.24
42	%	50.42 ± 3.52	45.22 ± 5.01	47.93 ± 3.35	46.32 ± 7.27
	Total count (×10^6^)	565.04 ± 118.09	600.74 ± 154.14	698.48 ± 169.09	724.81 ± 211.02

**Table 3:** The percentage and total count of CD3^+^ cells in the spleen of broiler chickens after in ovo injection of yolkin. The values are presented as mean ± SD; n = 8. The values in the same line with no common superscript letter differ significantly (*p* < 0.05).

Age (Days)		CONTROL	100 µg/Egg	10 µg/Egg	1 µg/Egg
1	%	17.20 ^b^ ± 4.31	27.13 ^a^ ± 7.13	32.51 ^a^ ± 6.36	26.66 ^a^ ± 4.65
	Total count (×10^6^)	0.91 ^b^ ± 0.26	1.43 ^ab^ ± 0.54	1.60 ^a^ ± 0.60	1.25 ^ab^ ± 0.38
7	%	51.73 ± 6.11	52.97 ± 8.98	50.74 ± 5.13	53.23 ± 2.71
	Total count (×10^6^)	43.83 ± 12.25	38.83 ± 8.82	35.40 ± 7.98	38.35 ± 13.99
14	%	50.29 ± 5.26	57.45 ± 9.99	57.66 ± 5.65	52.97 ± 3.05
	Total count (×10^6^)	144.42 ± 31.24	144.89 ± 35.53	123.52 ± 28.02	134.32 ± 14.72
21	%	42.94 ± 2.67	45.94 ± 4.56	42.87 ± 8.54	45.11 ± 3.15
	Total count (×10^6^)	190.13 ± 32.52	190.93 ± 40.39	177.96 ± 59.96	198.76 ± 45.07
28	%	42.44 ^a^ ± 2.14	41.43 ^a^ ± 3.17	37.19 ^b^ ± 3.07	39.86 ^ab^ ± 3.54
	Total count (×10^6^)	220.27 ± 36.47	219.86 ± 39.12	183.14 ± 36.61	187.35 ± 42.19
35	%	34.06 ^bc^ ± 4.34	31.24 ^c^ ± 7.17	39.02 ^ab^ ± 3.80	42.93 ^a^ ± 4.35
	Total count (×10^6^)	479.86 ± 105.62	457.30 ± 97.93	550.06 ± 105.02	526.23 ± 105.06
42	%	34.98 ± 2.93	41.85 ± 5.88	41.13 ± 4.35	41.92 ± 7.39
	Total count (×10^6^)	399.74 ^b^ ± 120.14	543.83 ^ab^ ± 91.72	593.47 ^a^ ± 115.65	638.91 ^a^ ± 127.80

**Table 4:** The percentage and total count of CD4^+^ cells in the spleen of broiler chickens after in ovo injection of yolkin. The values are presented as mean ± SD; n = 8. The values in the same line with no common superscript letter differ significantly (*p* < 0.05).

Age (Days)		CONTROL	100 µg/Egg	10 µg/Egg	1 µg/Egg
1	%	6.74 ^b^ ± 2.82	9.14 ^ab^ ± 3.79	12.90 ^a^ ± 4.89	11.41 ^ab^ ± 2.39
	Total count (×10^6^)	0.35 ± 0.14	0.48 ± 0.23	0.67 ± 0.40	0.54 ± 0.20
7	%	20.08 ± 3.37	20.24 ± 3.78	20.52 ± 5.12	20.78 ± 3.60
	Total count (×10^6^)	17.05 ± 5.39	14.92 ± 4.24	14.19 ± 4.13	14.90 ± 5.88
14	%	19.80 ± 2.32	22.93 ± 5.12	22.12 ± 5.59	21.12 ± 3.08
	Total count (×10^6^)	57.51 ± 16.10	57.59 ± 15.15	47.33 ± 14.48	53.89 ± 11.86
21	%	16.05 ± 2.13	16.46 ± 2.45	17.47 ± 4.15	19.74 ± 3.02
	Total count (×10^6^)	70.79 ± 12.81	68.32 ± 15.94	73.11 ± 30.41	88.08 ± 27.99
28	%	18.25 ^ab^ ± 2.25	16.35 ^b^ ± 2.12	15.58 ^b^ ± 3.23	20.53 ^a^ ± 3.16
	Total count (×10^6^)	94.11 ± 14.42	86.18 ± 12.24	77.16 ± 22.39	96.10 ± 22.11
35	%	12.93 ^bc^ ± 2.41	10.42 ^c^ ± 2.20	15.99 ^b^ ± 1.54	21.26 ^a^ ± 4.39
	Total count (×10^6^)	175.69 ^bc^ ± 51.56	155.28 ^c^ ± 43.80 b	225.23 ^ab^ ± 40.88	256.91 ^a^ ± 52.12
42	%	9.63 ^b^ ± 1.91	10.42 ^b^ ± 2.33	12.31 ^b^ ± 1.29	17.41 ^a^ ± 5.66
	Total count (×10^6^)	110.31 ^c^ ± 36.97	138.29 ^bc^ ± 50.60	180.31 ^b^ ± 47.01	259.08 ^a^ ± 63.38

**Table 5:** The percentage and total count of CD8^+^ cells in the spleen of broiler chickens after in ovo injection of yolkin. The values are presented as mean ± SD; n = 8. The values in the same line with no common superscript letter differ significantly (*p* < 0.05).

Age (Days)		CONTROL	100 µg/Egg	10 µg/Egg	1 µg/Egg
1	%	12.48 ^b^ ± 2.31	18.04 ^a^ ± 3.66	17.62 ^a^ ± 4.71	15.55 ^ab^ ± 3.57
	Total count (×10^6^)	0.67 ± 0.21	0.94 ± 0.26	0.85 ± 0.29	0.73 ± 0.24
7	%	32.74 ± 4.63	35.59 ± 6.11	29.98 ± 2.52	32.65 ± 2.43
	Total count (×10^6^)	27.58 ± 7.37	25.97 ± 5.77	21.09 ± 5.22	23.55 ± 8.71
14	%	28.01 ± 5.61	31.21 ± 6.28	33.63 ± 2.18	29.23 ± 5.66
	Total count (×10^6^)	79.03 ± 14.24	79.00 ± 22.49	71.99 ± 15.35	74.11 ± 15.07
21	%	22.31 ± 2.27	26.46 ± 4.73	23.99 ± 3.51	22.83 ± 2.81
	Total count (×10^6^)	99.19 ± 20.10	112.44 ± 36.99	99.20 ± 31.43	100.52 ± 24.30
28	%	23.31 ^ab^ ± 2.14	26.68 ^a^ ± 4.21	22.67 ^ab^ ± 3.54	21.88 ^b^ ± 2.81
	Total count (×10^6^)	120.33 ^ab^ ± 17.41	142.30 ^a^ ± 36.21	111.11 ^ab^ ± 25.91	103.07 ^b^ ± 25.55
35	%	20.52 ± 3.12	22.32 ± 3.76	23.12 ± 3.18	23.30 ± 3.22
	Total count (×10^6^)	278.95 ± 69.74	326.00 ± 43.98	330.04 ± 86.75	289.22 ± 84.91
42	%	23.69 ^b^ ± 3.77	30.17 ^a^ ± 4.60	29.42 ^a^ ± 4.82	26.95 ^ab^ ± 3.13
	Total count (×10^6^)	262.55 ^b^ ± 51.94	389.94 ^a^ ± 48.32	423.87 ^a^ ± 94.68	412.02 ^a^ ± 74.18

**Table 6:** The percentage and total count of KUL01^+^ cells in the spleen of broiler chickens after in ovo injection of yolkin. The values are presented as mean ± SD; n = 8. The values in the same line with no common superscript letter differ significantly (*p* < 0.05).

Age (Days)		CONTROL	100 µg/Egg	10 µg/Egg	1 µg/Egg
1	%	11.37 ^a^ ± 2.38	10.05 ^ab^ ± 1.78	8.09 ^b^ ± 2.24	9.62 ^ab^ ± 1.64
	Total count (×10^6^)	0.61 ± 0.17	0.52 ± 0.10	0.41 ± 0.21	0.46 ± 0.16
7	%	3.55 ^b^ ± 0.71	4.08 ^ab^ ± 1.06	4.91 ^a^ ± 0.84	3.61 ^b^ ± 0.55
	Total count (×10^6^)	2.95 ± 0.63	2.99 ± 0.94	3.53 ± 1.27	2.52 ± 0.74
14	%	2.69 ^a^ ± 0.33	1.90 ^b^ ± 0.49	1.88 ^b^ ± 0.36	2.56 ^a^ ± 0.42
	Total count (×10^6^)	7.87 ^a^ ± 2.49	4.74 ^b^ ± 1.34	4.10 ^b^ ± 1.36	6.59 ^ab^ ± 1.98
21	%	1.54 ± 0.59	1.73 ± 0.82	1.61 ± 0.45	1.63 ± 0.45
	Total count (×10^6^)	7.04 ± 3.89	7.07 ± 2.84	6.63 ± 2.58	6.93 ± 1.71
28	%	1.31 ^b^ ± 0.22	1.32 ^b^ ± 0.27	1.74 ^a^ ± 0.40	1.24 ^b^ ± 0.26
	Total count (×10^6^)	6.77 ^ab^ ± 1.54	6.90 ^ab^ ± 1.31	8.47 ^a^ ± 1.99	5.82 ^b^ ± 1.68
35	%	2.06 ^bc^ ± 0.34	3.04 ^a^ ± 1.02	2.62 ^ab^ ± 0.53	1.39 ^c^ ± 0.33
	Total count (×10^6^)	27.58 ^bc^ ± 5.88	44.90 ^a^ ± 17.31	37.41 ^ab^ ± 12.07	17.54 ^c^ ± 7.07
42	%	2.96 ± 0.41	3.01 ± 1.04	2.72 ± 0.67	3.47 ± 1.03
	Total count (×10^6^)	33.72 ± 10.61	40.95 ± 19.46	39.81 ± 14.43	53.20 ± 19.20

**Table 7:** The percentage and total count of Bu-1a^+^ cells in the blood of broiler chickens after in ovo injection of yolkin. The values are presented as mean ± SD; n = 8. The values in the same line with no common superscript letter differ significantly (*p* < 0.05).

Age (Days)		CONTROL	100 µg/Egg	10 µg/Egg	1 µg/Egg
1	%	56.20 ^a^ ± 12.40	55.89 ^a^ ± 5.90	43.92 ^b^ ± 7.90	49.93 ^ab^ ± 7.45
	Total count (×10^6^/mL)	7.89 ± 2.89	10.94 ± 2.88	11.31 ± 3.66	10.81 ± 2.33
7	%	55.82 ^b^ ± 9.68	66.44 ^a^ ± 7.50	58.37 ^ab^ ± 3.77	60.47 ^ab^ ± 7.23
	Total count (×10^6^/mL)	15.12 ^b^ ± 5.11	25.97 ^a^ ± 8.60	20.88 ^ab^ ± 3.07	23.85 ^ab^ ± 7.13
14	%	40.74 ± 10.85	33.04 ± 7.43	33.37 ± 14.03	30.63 ± 2.38
	Total count (×10^6^/mL)	8.33 ± 2.31	8.15 ± 2.96	9.20 ± 3.88	6.46 ± 1.20
21	%	36.49 ± 8.12	35.39 ± 7.82	36.52 ± 5.95	38.12 ± 6.71
	Total count (×10^6^/mL)	9.25 ± 2.15	10.76 ± 2.58	10.05 ± 3.41	9.35 ± 1.72
28	%	31.70 ^ab^ ± 4.32	29.75 ^b^ ± 2.84	33.76 ^ab^ ± 3.96	37.83 ^a^ ± 8.73
	Total count (×10^6^/mL)	15.19 ± 3.26	16.54 ± 2.30	17.07 ± 3.65	18.62 ± 4.27
35	%	26.12 ± 3.82	25.47 ± 3.22	28.20 ± 5.68	28.79 ± 3.90
	Total count (×10^6^/mL)	13.28 ^b^ ± 3.49	15.90 ^ab^ ± 2.59	16.75 ^ab^ ± 4.97	19.76 ^a^ ± 5.02
42	%	27.80 ± 4.63	26.18 ± 3.10	29.73 ± 7.18	25.84 ± 4.23
	Total count (×10^6^/mL)	14.91 ± 4.04	15.82 ± 2.07	17.08 ± 4.52	16.01 ± 3.65

**Table 8:** The percentage and total count of CD3^+^ cells in the blood of broiler chickens after in ovo injection of yolkin. The values are presented as mean ± SD; n = 8. The values in the same line with no common superscript letter differ significantly (*p* < 0.05).

Age (Days)		CONTROL	100 µg/Egg	10 µg/Egg	1 µg/Egg
1	%	13.08 ^b^ ± 3.70	16.94 ^ab^ ± 4.01	21.65 ^a^ ± 8.89	16.99 ^ab^ ± 4.97
	Total count (×10^6^/mL)	1.80 ^b^ ± 0.56	3.22 ^b^ ± 0.82	5.49 ^a^ ± 2.61	3.87 ^ab^ ± 1.70
7	%	17.83 ± 8.35	9.65 ± 6.01	13.30 ± 3.36	10.88 ± 5.77
	Total count (×10^6^/mL)	4.51 ± 1.94	3.33 ± 1.68	4.69 ± 1.04	3.92 ± 1.58
14	%	51.68 ± 9.55	52.87 ± 9.25	56.41 ± 14.94	58.98 ± 4.81
	Total count (×10^6^/mL)	10.52 ^b^ ± 2.12	12.60 ^ab^ ± 2.19	15.94 ^a^ ± 6.35	12.70 ^ab^ ± 3.51
21	%	52.50 ± 8.91	51.53 ± 7.84	51.57 ± 3.34	45.13 ± 7.42
	Total count (×10^6^/mL)	13.77 ± 5.14	15.96 ± 4.64	14.17 ± 3.73	11.52 ± 3.89
28	%	55.02 ± 6.85	57.03 ± 3.89	51.33 ± 5.13	50.85 ± 9.78
	Total count (×10^6^/mL)	26.23 ± 4.95	32.17 ± 7.43	26.06 ± 6.37	25.28 ± 6.38
35	%	50.30 ± 9.71	51.96 ± 9.20	43.34 ± 6.79	48.80 ± 6.15
	Total count (×10^6^/mL)	25.68 ± 7.71	32.93 ± 9.70	25.63 ± 6.18	32.81 ± 4.23
42	%	54.94 ^ab^ ± 7.54	60.40 ^a^ ± 4.60	51.38 ^b^ ± 6.97	59.55 ^ab^ ± 6.88
	Total count (×10^6^/mL)	29.06 ^c^ ± 5.51	36.76 ^a^ ± 6.06	29.34 ^bc^ ± 4.08	36.49 ^ab^ ± 5.23

**Table 9:** The percentage and total count of CD4^+^ cells in the blood of broiler chickens after in ovo injection of yolkin. The values are presented as mean ± SD; n = 8. The values in the same line with no common superscript letter differ significantly (*p* < 0.05).

Age (Days)		CONTROL	100 µg/Egg	10 µg/Egg	1 µg/Egg
1	%	9.52 ± 2.03	12.64 ± 3.57	14.22 ± 6.33	11.62 ± 4.27
	Total count (×10^6^/mL)	1.30 ^b^ ± 0.30	2.42 ^ab^ ± 0.83	3.62 ^a^ ± 1.84	2.61 ^ab^ ± 1.14
7	%	14.28 ± 6.22	8.29 ± 5.59	11.07 ± 3.92	8.69 ± 3.40
	Total count (×10^6^/mL)	3.61 ± 1.35	2.84 ± 1.56	3.92 ± 1.38	3.21 ± 1.06
14	%	39.03 ± 9.21	39.44 ± 4.68	42.88 ± 10.55	43.66 ± 7.84
	Total count (×10^6^/mL)	7.96 ^b^ ± 2.03	9.44 ^ab^ ± 1.50	12.04 ^a^ ± 4.39	9.43 ^ab^ ± 2.92
21	%	46.72 ± 10.73	46.61 ± 7.54	43.03 ± 3.41	38.80 ± 6.55
	Total count (×10^6^/mL)	12.29 ± 5.16	14.50 ± 4.51	11.87 ± 3.55	9.98 ± 3.53
28	%	45.21 ^ab^ ± 6.25	51.11 ^a^ ± 6.59	40.73 ^b^ ± 3.31	40.89 ^b^ ± 5.99
	Total count (×10^6^/mL)	21.31 ^b^ ± 2.49	28.96 ^a^ ± 8.15	20.47 ^b^ ± 3.29	20.33 ^b^ ± 4.48
35	%	40.96 ± 5.66	43.91 ± 4.64	36.67 ± 4.90	41.30 ± 8.62
	Total count (×10^6^/mL)	20.83 ± 5.20	27.51 ± 5.19	21.47 ± 3.86	28.03 ± 7.34
42	%	43.31 ± 6.68	45.17 ± 7.02	40.35 ± 8.05	50.03 ± 9.42
	Total count (×10^6^/mL)	22.81 ^b^ ± 3.75	27.36 ^ab^ ± 5.06	22.99 ^b^ ± 4.34	30.77 ^a^ ± 7.03

**Table 10:** The percentage and total count of CD8^+^ cells in the blood of broiler chickens after in ovo injection of yolkin. The values are presented as mean ± SD; n = 8. The values in the same line with no common superscript letter differ significantly (*p* < 0.05).

Age (Days)		CONTROL	100 µg/Egg	10 µg/Egg	1 µg/Egg
1	%	1.75 ^b^ ± 0.77	2.73 ^ab^ ± 0.66	2.95 ^a^ ± 0.97	2.43 ^ab^ ± 0.50
	Total count (×10^6^/^mL^)	0.24 ^b^ ± 0.10	0.51 ^a^ ± 0.11	0.74 ^a^ ± 0.27	0.55 ^a^ ± 0.23
7	%	4.38 ± 1.90	2.97 ± 2.36	3.71 ± 1.40	2.41 ± 1.09
	Total count (×10^6^/^mL^)	1.13 ± 0.50	1.05 ± 0.75	1.32 ± 0.50	0.89 ± 0.36
14	%	11.66 ^b^ ± 1.50	12.35 ^ab^ ± 2.77	14.57 ^ab^ ± 3.55	15.98 ^a^ ± 3.24
	Total count (×10^6^/^mL^)	2.38 ^b^ ± 0.43	2.91 ^ab^ ± 0.52	4.07 ^a^ ± 1.43	3.36 ^ab^ ± 0.82
21	%	9.95 ± 2.41	10.54 ± 1.16	11.97 ± 2.52	11.29 ± 2.16
	Total count (×10^6^/^mL^)	2.53 ± 0.67	3.26 ± 0.84	3.23 ± 0.87	2.83 ± 0.84
28	%	8.95 ^b^ ± 2.29	11.23 ^ab^ ± 2.37	9.81 ^ab^ ± 2.31	12.91 ^a^ ± 3.15
	Total count (×10^6^/^mL^)	4.38 ± 1.76	6.45 ± 2.48	4.84 ± 0.86	6.40 ± 1.83
35	%	12.33 ± 4.02	14.61 ± 4.09	12.96 ± 2.11	13.07 ± 1.86
	Total count (×10^6^/^mL^)	6.35 ± 2.73	8.96 ± 2.14	7.71 ± 1.91	8.87 ± 1.75
42	%	12.47 ^b^ ± 3.90	17.91 ^a^ ± 2.71	14.19 ^ab^ ± 2.71	12.78 ^b^ ± 4.31
	Total count (×10^6^/^mL^)	6.82 ^b^ ± 2.93	10.79 ^a^ ± 1.54	8.13 ^ab^ ± 1.79	7.84 ^ab^ ± 2.74

**Table 11:** The percentage and total count of KUL01^+^ cells in the blood of broiler chickens after in ovo injection of yolkin. The values are presented as mean ± SD; n = 8. The values in the same line with no common superscript letter differ significantly (*p* < 0.05).

Age (Days)		CONTROL	100 µg/Egg	10 µg/Egg	1 µg/Egg
1	%	4.74 ^b^ ± 1.84	11.39 ^ab^ ± 5.79	9.05 ^ab^ ± 3.71	14.88 ^a^ ± 6.79
	Total count (×10^6^/^mL^)	0.66 ^b^ ± 0.28	2.23 ^ab^ ± 1.38	2.37 ^ab^ ± 1.35	3.48 ^a^ ± 2.11
7	%	6.36 ^a^ ± 2.80	3.86 ^ab^ ± 1.26	2.59 ^b^ ± 1.24	2.30 ^b^ ± 2.02
	Total count (×10^6^/^mL^)	1.57 ^a^ ± 0.40	1.45 ^ab^ ± 0.45	0.94 ^ab^ ± 0.49	0.82 ^b^ ± 0.62
14	%	6.63 ± 3.96	5.06 ± 1.63	4.21 ± 1.36	7.13 ± 2.17
	Total count (×10^6^/^mL^)	1.34 ± 0.76	1.27 ± 0.57	1.19 ± 0.53	1.51 ± 0.57
21	%	6.91 ± 3.78	8.86 ± 4.23	7.36 ± 2.16	9.00 ± 3.88
	Total count (×10^6^/^mL^)	1.74 ± 0.86	2.67 ± 1.38	2.10 ± 1.13	2.12 ± 0.57
28	%	6.92 ± 1.26	4.31 ± 2.26	9.01 ± 4.63	9.27 ± 6.34
	Total count (×10^6^/^mL^)	3.33 ± 0.92	2.45 ± 1.36	4.71 ± 2.82	4.51 ± 2.95
35	%	8.40 ± 4.23	5.94 ± 3.92	9.20 ± 3.48	7.03 ± 3.64
	Total count (×10^6^/^mL^)	4.17 ± 2.06	3.81 ± 2.79	5.31 ± 1.72	4.83 ± 2.69
42	%	11.51 ^ab^ ± 5.68	7.55 ^b^ ± 3.18	15.26 ^ab^ ± 9.65	18.16 ^a^ ± 9.30
	Total count (×10^6^/^mL^)	6.08 ^ab^ ± 2.90	4.70 ^b^ ± 2.17	8.84 ^ab^ ± 5.72	11.25 ^a^ ± 6.15

## Data Availability

Data are contained within the article and the Supplementary Materials.

## Supplementary Materials

The following supporting information can be downloaded at: https://www.mdpi.com/article/10.3390/ijms242417494/s1.
