# Supplementary material for: Yolkin, a Polypeptide Complex from Egg Yolk, Affects Cytokine Levels and Leukocyte Populations in Broiler Chicken Blood and Lymphoid Organs after In Ovo Administration
Source: Int J Mol Sci. 2023 Dec 14;24(24):17494. doi: 10.3390/ijms242417494 (PMC10743580; doi:10.3390/ijms242417494)
Supplement: Supplementary file 1 [file ijms-24-17494-s001.zip › ijms-2750139-supplementary.pdf]

Table S1. The percentage and total count of $CD4^{-}CD8^{-}$ cells in the thymus of chicken broilers after *in ovo* injection of yolkin. The values are presented as mean ± SD; n=8.
The values in the same line with no common superscript letter differ significantly (p<0.05).

Age (days)		CONTROL	100 µg/egg	10 µg/egg	1 µg/egg
1		not tested	not tested	not tested	not tested
7	%	7.90^{b} ±1.44	12.76^{a} ±3.47	9.40^{b} ±1.25	10.13^{ab} ±2.01
	Total count (x10^6)	27.34^{b} ±5.02	44.94^{a} ±13.94	30.29^{b} ±5.67	36.97^{ab} ±7.82
14	%	6.48 ±2.68	11.01 ±6.00	7.26 ±1.83	6.55 ±1.48
	Total count (x10^6)	44.66 ±15.55	70.73 ±43.10	43.13 ±14.47	48.65 ±15.95
21	%	6.55 ±2.27	8.75 ±2.48	9.32 ±1.74	8.57 ±4.04
	Total count (x10^6)	60.41 ±17.82	81.33 ±27.53	94.42 ±11.94	80.63 ±33.48
28	%	9.33 ±2.06	10.18 ±1.57	11.99 ±3.01	10.40 ±2.90
	Total count (x10^6)	156.20 ±40.77	170.15 ±51.86	206.17 ±79.19	159.77 ±64.66
35	%	8.17 ±2.37	8.90 ±1.61	6.85 ±1.90	11.69 ±6.28
	Total count (x10^6)	176.99 ±64.24	177.92 ±26.32	119.96 ±46.89	183.08 ±105.58
42	%	8.46 ±2.86	7.19 ±2.81	7.03 ±2.52	7.53 ±3.01
	Total count (x10^6)	197.80 ±89.21	165.42 ±69.01	152.13 ±47.07	149.80 ±71.02

Table S2. The percentage and total count of $CD4^+$ cells in the thymus of chicken broilers after *in ovo* injection of yolkin. The values are presented as mean ± SD; n=8

The values in the same line with no common superscript letter differ significantly ($p<0.05$).

Age (days)		CONTROL	100 µg/egg	10 µg/egg	1 µg/egg
1		not tested	not tested	not tested	not tested
7	%	1.69 ±0.86	1.81 ±0.77	1.27 ±0.32	1.41 ±0.39
	Total count ($x10^6$)	6.25 ±4.11	6.36 ±2.74	4.09 ±1.15	5.22 ±1.60
14	%	2.23 ±0.71	2.37 ±1.12	2.91 ±2.10	1.53 ±0.38
	Total count ($x10^6$)	15.35 ±4.18	14.97 ±7.87	16.94 ±11.75	11.33 ±3.68
21	%	1.69 ±0.75	1.49 ±0.24	1.46 ±0.70	1.76 ±0.40
	Total count ($x10^6$)	16.40 ±8.92	14.09 ±3.98	14.48 ±5.32	16.77 ±3.68
28	%	1.38 ±0.24	1.43 ±0.43	1.18 ±0.47	1.40 ±0.45
	Total count ($x10^6$)	23.04 ±5.22	23.76 ±8.76	19.73 ±7.41	21.50 ±9.90
35	%	2.09^{ab} ±0.34	1.58^{b} ±0.38	2.73^{a} ±1.44	2.35^{ab} ±0.58
	Total count ($x10^6$)	45.92 ±12.80	31.40 ±5.80	48.63 ±28.21	37.11 ±11.85
42	%	1.54 ±0.41	1.72 ±0.39	2.17 ±0.66	1.61 ±0.58
	Total count ($x10^6$)	36.19 ±12.74	40.03 ±9.11	46.98 ±14.15	31.80 ±13.79

Table S3. The percentage and total count of $CD8^+$ cells in the thymus of chicken broilers after *in ovo* injection of yolkin. The values are presented as mean ± SD; n=8

The values in the same line with no common superscript letter differ significantly ($p<0.05$).

Age (days)		CONTROL	100 µg/egg	10 µg/egg	1 µg/egg
1		not tested	not tested	not tested	not tested
7	%	2.49^{ab} ±1.09	3.43^{a} ±1.49	1.70^{b} ±0.70	2.09^{ab} ±0.99
	Total count ($x10^6$)	8.73^{ab} ±4.06	12.04^{a} ±5.34	5.47^{b} ±2.31	7.97^{ab} ±5.09
14	%	5.34 ±1.60	5.45 ±2.21	5.17 ±1.66	6.33 ±1.36
	Total count ($x10^6$)	38.03 ±14.08	33.68 ±13.94	31.01 ±14.41	46.19 ±11.42
21	%	4.91^{ab} ±0.97	7.66^{a} ±2.75	4.33^{b} ±1.42	5.92^{ab} ±2.84
	Total count ($x10^6$)	46.37 ±11.24	72.00 ±31.38	44.95 ±17.02	56.19 ±24.12
28	%	5.47^{ab} ±1.24	4.93^{ab} ±1.58	3.80^{b} ±1.02	5.85^{a} ±1.04
	Total count ($x10^6$)	91.46 ±24.82	78.92 ±18.26	62.74 ±13.66	90.40 ±33.02
35	%	5.17 ±1.73	6.41 ±1.88	7.29 ±1.13	7.22 ±2.12
	Total count ($x10^6$)	112.19 ±40.55	129.86 ±37.59	124.40 ±34.10	114.62 ±38.66
42	%	5.56 ±1.09	5.93 ±1.83	7.47 ±3.12	6.73 ±2.67
	Total count ($x10^6$)	133.48 ±50.82	142.73 ±58.90	164.84 ±75.70	129.19 ±53.78

Table S4. The $CD4^+/CD8^+$ ratio of cells in the thymus of chicken broilers after *in ovo* injection of yolkin. The values are presented as mean ± SD; n=8

The values in the same line with no common superscript letter differ significantly (p<0.05).

Age (days)	CONTROL	100 µg/egg	10 µg/egg	1 µg/egg
1	not tested	not tested	not tested	not tested
7	0.76 ±0.48	0.68 ±0.48	0.84 ±0.29	0.83 ±0.45
14	0.45 ±0.22	0.66 ±0.76	0.58 ±0.41	0.26 ±0.10
21	0.35 ±0.17	0.22 ±0.09	0.35 ±0.20	0.36 ±0.18
28	0.27 ±0.10	0.33 ±0.21	0.32 ±0.09	0.24 ±0.06
35	0.43 ±0.13	0.27 ±0.14	0.38 ±0.19	0.37 ±0.18
42	0.29 ±0.11	0.31 ±0.12	0.33 ±0.15	0.26 ±0.12

Table S5. The $CD4^+/CD8^+$ ratio of cells in the spleen of chicken broilers after *in ovo* injection of yolkin. The values are presented as mean ± SD; n=8

The values in the same line with no common superscript letter differ significantly (p<0.05).

Age (days)	CONTROL	100 µg/egg	10 µg/egg	1 µg/egg
1	0.54 ±0.23	0.50 ±0.13	0.83 ±0.54	0.54 ±0.23
7	0.62 ±0.13	0.60 ±0.21	0.70 ±0.22	0.64 ±0.13
14	0.73 ±0.17	0.74 ±0.10	0.66 ±0.17	0.77 ±0.28
21	0.72^{ab} ±0.06	0.64^{b} ±0.16	0.74^{ab} ±0.20	0.88^{a} ±0.17
28	0.79^{ab} ±0.16	0.63^{b} ±0.16	0.72^{ab} ±0.27	0.96^{a} ±0.23
35	0.64^{b} ±0.11	0.47^{b} ±0.10	0.70^{b} ±0.12	0.94^{a} ±0.29
42	0.41^{b} ±0.09	0.35^{b} ±0.09	0.43^{b} ±0.09	0.64^{a} ±0.17

Table S6. The $CD4^+/CD8^+$ ratio of cells in the blood of chicken broilers after *in ovo* injection of yolkin. The values are presented as mean ± SD; n=8

The values in the same line with no common superscript letter differ significantly ($p<0.05$).

Age (days)	CONTROL	100 µg/egg	10 µg/egg	1 µg/egg
1	6.00 ±1.77	4.73 ±1.13	4.91 ±1.96	4.76 ±1.32
7	3.35 ±0.54	3.00 ±1.10	3.14 ±0.97	3.79 ±1.13
14	3.35 ±0.73	3.28 ±0.52	2.98 ±0.45	2.89 ±0.94
21	5.16 ±2.22	4.44 ±0.68	3.73 ±0.77	3.54 ±0.87
28	5.42^{a} ±1.80	4.74^{ab} ±1.15	4.33^{ab} ±0.92	3.28^{b} ±0.69
35	3.58 ±1.01	3.19 ±0.87	2.93 ±0.80	3.26 ±0.98
42	3.75^{ab} ±1.11	2.57^{b} ±0.58	2.97^{ab} ±0.92	4.17^{a} ±1.19

Table S7. Nutrient composition of starter, grower and finisher diets for chicken broilers.

Type	Starter	Grower		Finisher
Name	KW1	KW2	KW3	KW4
Age (days)	0-10	11-25	26-34	>34
Crude protein [%]	22.2	20.5	19.5	18.5
Raw fiber[%]	3	3.2	3	3.2
Fat [%]	4.4	4.6	6.5	7.6
Ash [%]	4.8	4.6	4	4
Lysin [%]	1.3	1.2	1.15	1.1
Metionin [%]	0.6	0.56	0.54	0.5
Calcium [%]	0.8	0.7	0.6	0.55
Sodium [%]	0.14	0.13	0.14	0.13
Phosphorus [%]	0.6	0.55	0.52	0.45

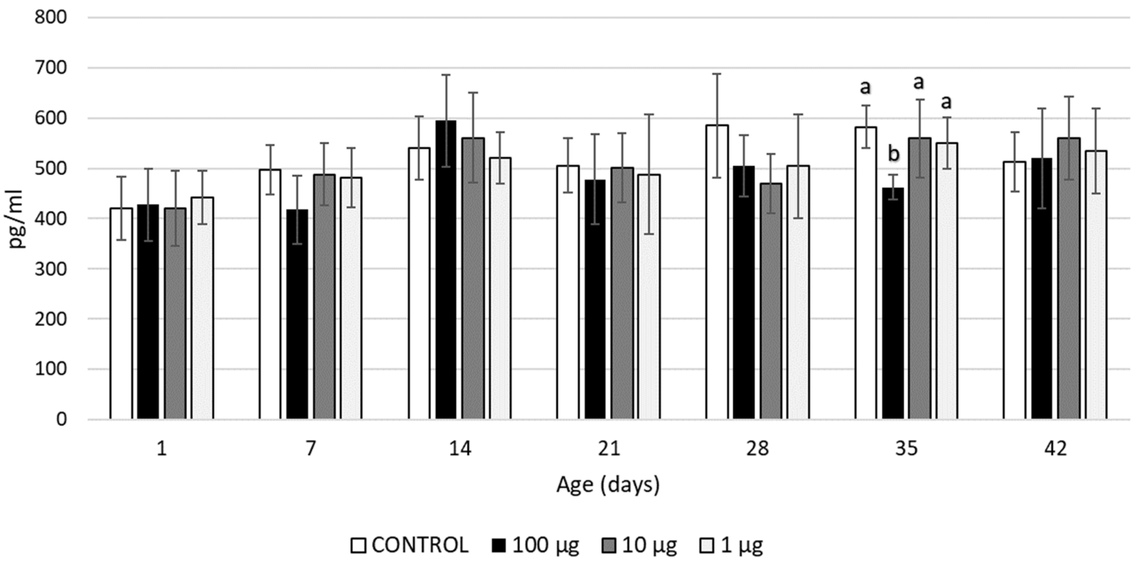

Figure S1. IL-6 levels (pg/ml) in the blood of chicken broilers after *in ovo* injection of yolkin. The values are presented as mean ± SD; n=8. The values within the same age group with no common superscript letter differ significantly (p<0.05).

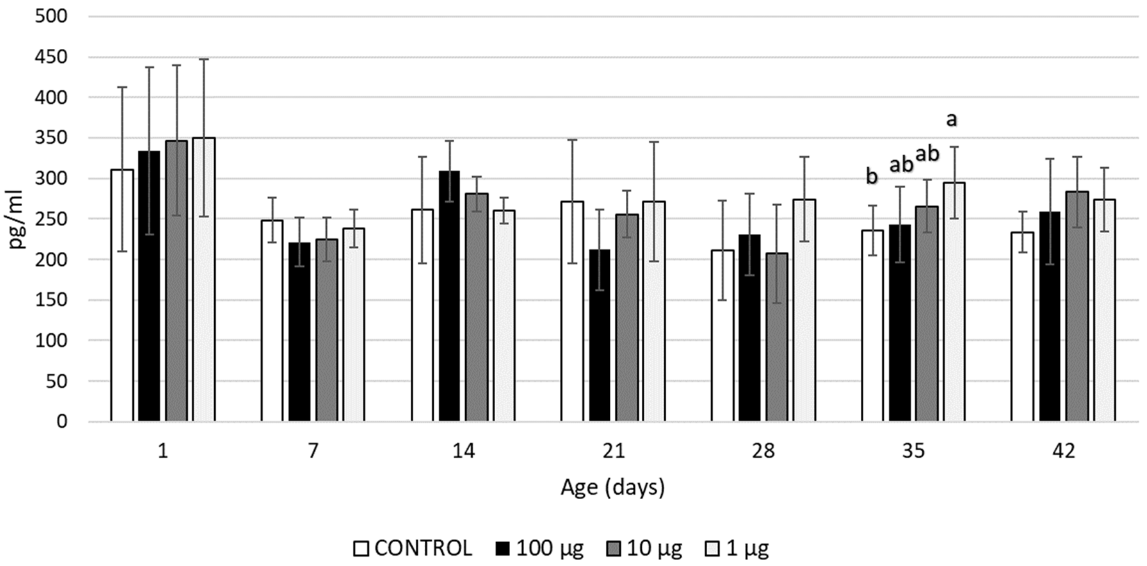

Figure S2. IL-10 levels (pg/ml) in the blood of chicken broilers after *in ovo* injection of yolkin. The values are presented as mean ± SD; n=8. The values within the same age group with no common superscript letter differ significantly (p<0.05).
